# Detecting Psychological Interventions Using Bilateral Electromyographic Wearable Sensors

**DOI:** 10.3390/s24051425

**Published:** 2024-02-22

**Authors:** Yedukondala Rao Veeranki, Sergi Garcia-Retortillo, Zacharias Papadakis, Andreas Stamatis, Kwadwo Osei Appiah-Kubi, Emily Locke, Ryan McCarthy, Ahmed Ali Torad, Ahmed Mahmoud Kadry, Mostafa Ali Elwan, Ali Boolani, Hugo F. Posada-Quintero

**Affiliations:** 1 Department of Biomedical Engineering, University of Connecticut, Storrs, CT 06269, USA; yedukondala_rao.veeranki@uconn.edu; 2 Department of Health and Exercise Science, Wake Forest University, Winston-Salem, NC 27109, USA; sgarcia@wfu.edu; 3 College of Health and Wellness, Barry University, Miami Shores, FL 33168, USA; zpapadakis@barry.edu; 4 Health and Sport Sciences, University of Louisville, Louisville, KY 40292, USA; coach_stam@rocketmail.com; 5 Sports Medicine Institute, University of Louisville Health, Louisville, KY 40208, USA; 6 Department of Physical Therapy, Clarkson University, Potsdam, NY 13699, USA; kappiahk@clarkson.edu (K.O.A.-K.); ahmed_alimohamed@pt.kfs.edu.eg (A.A.T.); ahmed_tabia@pt.kfs.edu.eg (A.M.K.); mostafa.ali@pt.bsu.edu.eg (M.A.E.); 7 Department of Public Health, Yale University, New Haven, CT 06520, USA; lockeee@clarkson.edu; 8 Department of Biology, Clarkson University, Potsdam, NY 13699, USA; mccartrm@clarkson.edu; 9 Department of Psychology, Clarkson University, Potsdam, NY 13699, USA; 10 Faculty of Physical Therapy, Kafrelsheik University, Kafr El Sheik 33516, Egypt; 11 Faculty of Physical Therapy, Beni-Suef University, Beni-Suef 62521, Egypt; 12 Department of Aeronautical and Mechanical Engineering, Clarkson University, Potsdam, NY 13699, USA; aboolani@clarkson.edu

**Keywords:** surface electromyography, wearable sensors, psychological interventions, auditory stimuli, classification

## Abstract

This study investigated the impact of auditory stimuli on muscular activation patterns using wearable surface electromyography (EMG) sensors. Employing four key muscles (Sternocleidomastoid Muscle (SCM), Cervical Erector Muscle (CEM), Quadricep Muscles (QMs), and Tibialis Muscle (TM)) and time domain features, we differentiated the effects of four interventions: silence, music, positive reinforcement, and negative reinforcement. The results demonstrated distinct muscle responses to the interventions, with the SCM and CEM being the most sensitive to changes and the TM being the most active and stimulus dependent. Post hoc analyses revealed significant intervention-specific activations in the CEM and TM for specific time points and intervention pairs, suggesting dynamic modulation and time-dependent integration. Multi-feature analysis identified both statistical and Hjorth features as potent discriminators, reflecting diverse adaptations in muscle recruitment, activation intensity, control, and signal dynamics. These features hold promise as potential biomarkers for monitoring muscle function in various clinical and research applications. Finally, muscle-specific Random Forest classification achieved the highest accuracy and Area Under the ROC Curve for the TM, indicating its potential for differentiating interventions with high precision. This study paves the way for personalized neuroadaptive interventions in rehabilitation, sports science, ergonomics, and healthcare by exploiting the diverse and dynamic landscape of muscle responses to auditory stimuli.

## 1. Introduction

Walking is the most common form of ambulation among non-disabled humans and is a part of most activities of daily life [1,2]. Human walking gait, although seemingly simple, is a complex symphony of muscle activation orchestrated by the brain [3]. Walking requires the coordinated action of 28 major muscles to counter gravity and propel the body forward by joint trunk and limb movement coordination to generate force that requires minimum energy expenditure [4,5]. These muscle activities are often measured in human walking gait using surface electromyography (sEMG), a non-invasive technique that measures the electrical output of muscles on the skin [6,7,8]. sEMG studies have identified significant inter-individual variability in motor coordination but limited intra-individual variability [6,7,8]. Taken together, these findings suggest that while different individuals have different gait patterns and motor coordination, gait and motor coordination for an individual does not vary from time point to time point.

Studies that have tried to identify within-subject changes in motor coordination patterns use either physical tasks to change motor coordination [9,10] or use a dual-task paradigm to modify gait and muscle activity patterns [11,12]. The dual-task paradigm for modifying gait and muscle activity pattern focuses on disrupting various motor and cognitive processes, including attention to the environment and neurobiomechanical control of the trunk and limbs during locomotion [13,14,15]. These findings [13,14,15], combined with the works of various scholars, on the effects of various moods, such as anxiety [16,17], depression [18,19,20], anger [21], and fatigue [22,23,24,25], suggest that psychological conditions can disrupt motor coordination patterns.

Interestingly, regarding the studies that have used psychological interventions to modify muscle activity, their findings have been contradictory [26,27]. For example, in a study that induced feelings of anxiety in trumpet players, the authors reported a significant modification of muscle activity and increase in muscle fatigue [26]. Conversely, in a study that sought to create unpleasant feelings among their participants, no significant impact on muscle activity was reported [27]. These contradictory findings suggest that the role of modifying feelings in muscle activity is unclear. One potential difference in the results of these studies may be the fact that Rumsey and colleagues [26] gave one-time auditory instructions to induce anxiety, while D’Attilio and colleagues [27] measured sEMG changes during continuous visual input meant to modify affect. Another potential difference between the two studies is that Rumsey and colleagues [26] assessed changes in motor activation during dynamic movement, while D’Attilio and colleagues [27] assessed changes in motor activation pattern during static posture.

Therefore, to assess the impact of psychological interventions on muscle activation patterns, the authors of this study chose to implement auditory psychological interventions on a dynamic movement, specifically walking gait. Due to the limited intra-individual variability of walking gait [6,7,8] and previous work supporting the impact of affect on modifying walking gait [16,17,18,19,20,21], the authors chose to use walking gait to assess the impact of various psychological interventions on walking gait muscle firing patterns. Also, based on the previous works of these authors on the impact of music, silence, positive, and negative feedback on modifying basketball jump shooting performance [28], the authors chose to use these four interventions to modify muscle activation patterns. Thus, the objective of this study was to assess the impact of silence, the participant’s favorite music, and positive and negative feedback on muscle activation patterns during a 30 min walk. 

## 2. Methodology

### 2.1. Experimental Protocol

#### 2.1.1. Study Design

A randomized controlled, within-participant, crossover design was used to examine the effects of four conditions—silence, self-selected favorite music, negative feedback, and positive feedback—on moods, individual muscle firing patterns, and motor coordination. In all interventions, participants completed six 5 min walking segments, with participants’ moods being measured at baseline and after each 5 min walk. Participants completed each condition in a randomized order after the completion of a familiarization session. On average, participants began the study 3.4 ± 2.1 days after the familiarization day.

#### 2.1.2. Screening

Participants were recruited using campus-wide emails and verbal announcements in large classes (>20 students) at a small private university in northern New York and through the posting of flyers with QR codes throughout the campus. All interested participants were directed to an online survey to assess inclusion/exclusion criteria. To be included in this study, participants had to be able to stand and walk without an assistive device for a minimum of 60 min and had to be between the ages of 18 and 45. Participants were excluded if they had an impairment or were unable to perform physical activity independently, reported pain or discomfort when walking, had a neurological condition, had a recent (<6 months) orthopedic surgery that impacted their walking ability or balance, reported a wound on the plantar surface of their foot, or had a visual impairment.

#### 2.1.3. Participants

Approval for this study was granted by the Clarkson University Institutional Review Board (IRB) (approval #20.20-6). Volunteers not excluded by the screening questionnaire were invited to participate in the study and were scheduled for a familiarization day. At the beginning of the familiarization day, all participants read and initialed each page and signed the IRB-approved informed consent forms. Participants were informed that they would be participating in a study assessing the impact of various psychological interventions on moods and walking. 

The study was started in January 2020. However, due to the COVID-19 pandemic, all data collection was paused due to COVID-19 restrictions, and all data collected prior to March 2020 were excluded. Data collection restarted in July 2021 after all COVID restrictions had been lifted, and data collection was completed in May 2022. Therefore, the data reported here refer only to individuals who completed the study after July 2021. Of the 40 participants who completed the inclusion/exclusion survey after 8 July 2021 did not qualify for the study (all reported a lower extremity orthopedic injury). Of the 32 participants who qualified for the study, 26 completed the study (10 males, 16 female, age = 23.04 ± 5.64). The demographic characteristics of the participants are shown in Table 1. Using G*Power (version 3.1.9.6, Heinrich-Heine-Universitat Dusseldorf, Dusseldorf, Germany), an a priori power analysis was completed, and this analysis showed that a sample size of 20 would provide sufficient statistical power (α = 0.05, 1 − β = 0.80) to detect a 4-intervention × 7-time interaction effect size of 0.25, assuming a correlation across repeated measures on time of 0.50. To reduce the potential for Type II errors, 26 participants completed the study in case there were outliers and data had to be excluded.

#### 2.1.4. Auditory Stimulation for Four Interventions

1. Silence: Participants were instructed to wear noise-canceling headphones to eliminate external auditory stimuli during the walking sessions.
2. Music: Participants were allowed to choose and listen to music of their preference via a music streaming application of their choice, such as Amazon Music, Pandora, Spotify, iHeartRadio, or Apple Music, during the walking sessions.
3. Positive Reinforcement: Participants received auditory feedback in the form of positive reinforcement through the headphones every 30 s.
4. Negative Reinforcement: Participants received negative reinforcement through the headphones every 30 s.

Table 2 presents positive and negative interventions utilized in the study, including affirmations such as “Good job, you’re doing awesome!” and criticisms like “You’ve got to walk faster than that”.

#### 2.1.5. Experimental Procedure

Familiarization Day: If a subject met the inclusion/exclusion criteria, they were invited to the laboratory for a familiarization day. At the beginning of the familiarization day, all participants were informed that they would be asked to walk under four different conditions that they would be assigned to in random order. Participants were then told that they would have to wear noise-canceling headphones during all conditions and that, one day, they would be asked to walk in silence (silence), another day, they’d be asked to play their favorite music (music), another day, they would hear positive phrases through the headphones (positive feedback), and the final intervention would involve a research assistant screaming negative phrases at them through the headphones (negative feedback). All participants agreed to these conditions and signed the informed consent form.

**Figure 1:**
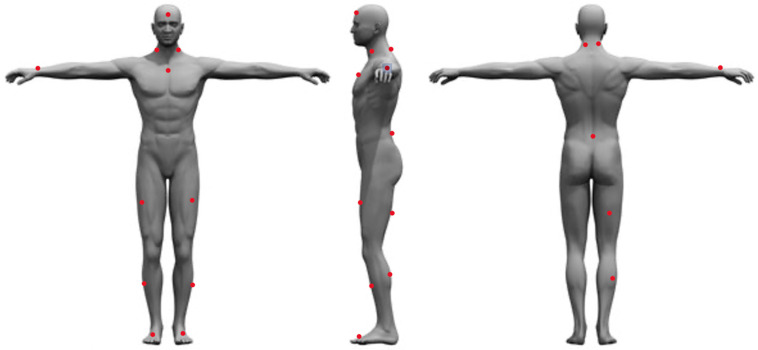
Sensor placement locations.

After signing the informed consent form, each participant’s height was measured using a stadiometer (SECA model 220, SECA Corporation, Chino, CA, USA), and weight was measured using the Tanita Bioelectrical Impedance Analysis Scale (TBF-410, Tanita Corporation, Tokyo, Japan). After height and weight data were collected, the subjects were asked to complete a series of surveys to assess baseline data such as mental traits and physical energy and fatigue, grit, sleep quality and quantity over the last 30 days, diet quality, moods, and physical activity behavior over the last 7 days. After the completion of these surveys, the participants were fitted with 16 Delsys Trigno sEMG/IMU sensors (Delsys Inc., Boston, MA, USA). The Delsys Trigno series comprises wireless, wearable sensors integrating sEMG and inertial measurement unit (IMU) functionalities. This technology enables the simultaneous capture of high-fidelity muscle activity and movement data, offering valuable insights for diverse applications. Available in configurations ranging from a single channel (Trigno Avanti) to up to 12 channels (Trigno Pro), these sensors boast high data quality, portability, and user comfort, making them well suited for biomechanics research, rehabilitation monitoring, sports performance analysis, and prosthetic/orthotic control studies. Prior to placing the sensors on the participant, the area of sensor placement was cleaned with alcohol wipes, then shaved using a disposable razor, cleaned again using an alcohol wipe and gauze, and then marked with a red washable marker. The sensors were then placed in the following locations: the forehead (between the frontal eminence and line with the nose); left and right Sternocleidomastoid (midpoint between the mastoid process and the clavicle); sternum (right below the notch of the manubrium); right wrist (between the ulna and radius on the last crest of the skin); left and right rectus femoris (three quarters of the distance between the greater trochanter and the superior aspect of the patella); left and right Tibialis Anterior (midpoint between the inferior aspect of the patella and the talo-tibial joint); left and right foot (outside of the shoe on the inner edge of the big toe); left and right Cervical Erector Spinae (C4), top of the iliac crest (on L4, right above L5-S1); belly of right bicep femoris longus (located by palpating the popliteal fossa and then moving proximally towards the ischial tuberosity to locate the muscle belly); and the belly of the right medial gastrocnemius (midpoint between popliteal fossa and the triceps surae) (Figure 1).

**Figure 2:**
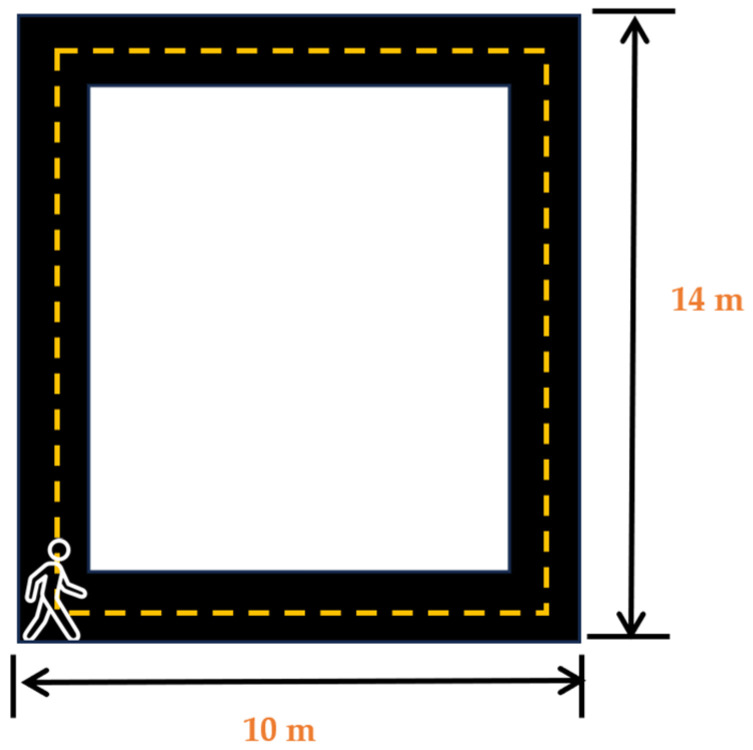
Walking track layout for familiarization and testing days.

After the placement of the sensors, the participants were asked wear noise-canceling headphones (COWIN E7 Active Noise Cancelling Headphones, Cowin Audio, City of Industry, CA, USA) and asked to play their favorite music or station on their favorite streaming application on the lab-provided Samsung Galaxy S5 (Samsung Electronics, Suwon, republic of Korea) wireless phone. Participants were informed that this was the music/station that would be selected for their music day trial. After participants had entered their choice, they were asked to stand at the start of the 14 m × 10 m walking track (Cone 1-Figure 2). Participants were then asked to complete a survey on a 12.9-inch iPad Pro (256 GB, model MLOT2LL/A) that asked them how they felt and how motivated they were to perform mental and physical tasks at the moment. After the completion of the survey, the participants were then asked to commence walking around the track towards cone 2, then turn towards cone 3, then towards cone 4, and then walk back towards cone 1. The participants were asked to walk at a speed that they felt comfortable walking at and were told that they would be walking for 5 min. After the completion of the 5 min walk, the participants were asked to complete another survey to ask them how they felt and how motivated they were to complete mental and physical tasks at that moment. Upon the completion of these surveys, the sensors were removed, and the participants were instructed to abstain from caffeine consumption and vigorous and moderate physical activity for a minimum of 12 h prior to their next session and to get their usual night’s amount of sleep. No data from the familiarization day were included in our analysis.

Testing Days 2–5: Participants were scheduled for 4 testing sessions, with each session being a minimum of 48 h apart but within 14 days of the previous session. The number of days between sessions was 4.2 ± 3.28 days. To limit the effects of diurnal variations, participants were scheduled ± 30 min from the time of their first testing day (i.e., if the first testing day was scheduled at 9:00, then the other 3 testing days were scheduled between 8:30 and 9:30) [29]. Since sleep has a substantial impact on gait [30], participants who reported 2 h more or less than their usual sleep duration (as self-reported on familiarization day) were not tested that day and were rescheduled. Participants who reported consuming caffeine and/or participating in moderate or vigorous physical activity 12 h prior to testing were also rescheduled. Using randomizer.org, the participants were randomly assigned their intervention order.

**Figure 3:**
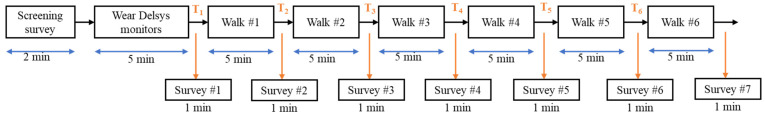
Experimental design: 30-minute walking intervention (carried out with 6 5-minute rounds) and pre/post-intervention surveys.

On each testing day, the participants came into the lab where they completed the pre-testing questionnaire to determine their testing eligibility. Using the same procedures and placement sites as the familiarization day, the participants were fitted with sensors. The participants were then asked to place the noise-canceling headphones on their ears and to go to the start of the walking track (cone 1; Figure 2). The participants were then informed what intervention they would be receiving and then asked to complete a series of surveys that assessed their current mood states and their motivation to perform mental and physical tasks. After the completion of these surveys, the auditory intervention was started, and participants began walking. The participants walked for 5 min prior to being asked to stop in place and complete a series of surveys that assessed their moods and motivation. The participants completed a series of 6 rounds of walking for a total of 30 min (see Figure 3). After the final round, the participants were again asked about their moods and motivation. The sensors were then removed, and the participants were again reminded of the pre-testing instructions and scheduled for their next session.

### 2.2. Pre-Processing of Recorded EMG Signals

#### 2.2.1. Filtering

Prior to further processing, the EMG signals were subjected to band-pass filtering between 20 Hz and 400 Hz using a digital Butterworth filter. This filtering step aimed to isolate the EMG signal from low-frequency noise, such as baseline drift or movement artifacts, and high-frequency artifacts, such as power line noise, thereby enhancing the signal-to-noise ratio and facilitating an accurate analysis of muscle activity.

#### 2.2.2. Resampling

To ensure compatibility with downstream analysis tools and software packages, the EMG signals were resampled from their original sampling frequency of 1059 Hz to a standard sampling frequency of 1200 Hz using a sinc interpolation method. Resampling is a common practice in data processing, particularly when dealing with signals from different sources that may have varying sampling rates. This step helps to synchronize the EMG signals with other data streams, such as motion capture or force plate data, enabling a more comprehensive analysis of gait and balance patterns.

#### 2.2.3. Segment Removal

To identify and remove segments of data that did not meet specific quality criteria, a validity check was performed. Segments with continuous zeros, indicating EMG signal absence, were considered invalid and marked for removal. Similarly, segments containing NaN (Not a Number) values, suggesting data corruption or missing values, were also identified and eliminated. Additionally, segments that exceeded a duration of 0.1 s were deemed invalid, as they might represent periods of excessive noise or signal disruption. These invalid segments were removed from the EMG data to ensure the integrity and reliability of the remaining data for further analysis.

#### 2.2.4. Handling Short Segments with Spline Interpolation

In instances where the validity check resulted in shorter segments, spline interpolation was employed to fill in the missing data points. Spline interpolation is a mathematical technique that constructs a smooth curve through a set of data points, allowing for the estimation of values at intermediate points. By applying spline interpolation to shorter segments, the continuity of the EMG signals was maintained, preventing abrupt transitions or gaps in the data. This approach proved particularly useful when dealing with segments that were shorter than the specified threshold of 0.1 s, enabling the preservation of valuable information despite the presence of minor data gaps.

### 2.3. Feature Extraction

Features, namely, statistical and Hjorth features, were extracted from the pre-processed EMG signals for a comprehensive analysis. A list of the extracted features and descriptions for each one are shown in Table 3.

### 2.4. Statistical Analysis and Classification

The extracted features were analyzed using a Repeated Measures ANOVA (RM ANOVA) [34] to determine the statistical significance of the time point pairs and intervention pairs. An RM ANOVA incorporates the non-independence of observations within subjects through a variance–covariance matrix, explicitly modeling the correlations among the repeated measures. Further, post hoc analysis was performed to explore where exactly the differences lie in the time point and intervention pairs [35,36]. Bonferroni correction was applied to the significance levels for these multiple comparisons [37]. Thus, the post hoc analysis provided more insights into the specific patterns of the features and allowed us to understand which time point/intervention pairs differ from each other. Also, the best feature for differentiating intervention and time point pairs for each muscle was computed using the select k best feature selection method.

The features were fed to a Random Forest (RF) classifier [38] for the classification of the 4 interventions. RF is an ensemble learning method that is based on combining multiple classifiers to handle a complex problem and improve performance. It aggregates the results of several decision trees on distinct subsets of a dataset to increase the dataset prediction accuracy. The more trees in the forest, the more accurate it is, and the risk of overfitting is minimized. The performance of the classifier was further evaluated using the following performance metrics (described in Table 4): accuracy (Acc), precision (Pre), recall (Rec), F1-score (F1), Specificity (spe), and Area Under the ROC Curve (AUC) [39].

## 3. Results

### 3.1. Representative EMG Signals during Four Psychological Interventions

**Figure 4:**
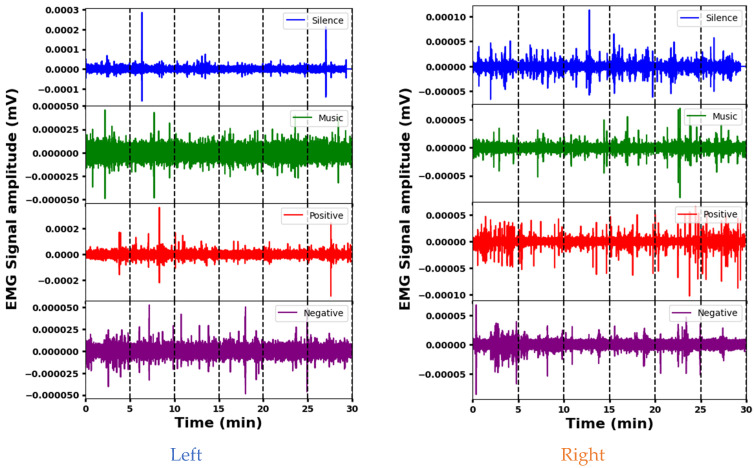
Representative EMG signals acquired from the Sternocleidomastoid muscle demonstrating variations across different psychological interventions.

The representative EMG signals acquired from the Sternocleidomastoid muscle in four psychological interventions are shown in Figure 4. The amplitudes of the EMG signals indicate the varying effects of the interventions (silence, music, and positive and negative stimuli) on left Sternocleidomastoid muscle activity. The mean amplitude is lowest during silence, indicating decreased muscle activity, while positive stimuli elicit the highest mean amplitude, indicating increased muscle engagement. The standard deviation shows that the variability differs between the signals, with positive and silence stimuli showing more variability compared to the music and negative interventions. The amplitude ranges also vary, with positive and negative stimuli having wider ranges, indicating increased muscle activity or signal fluctuations that may be associated with stress or psychological responses. Each intervention elicits different muscle engagement, signal strength, or psychological responses. A segment-wise analysis of the EMG data reveals distinct patterns. During silence, different amplitudes are observed across segments. Segment 6 is indicative of potentially increased muscle activity. Music elicits greater initial variability (segment 1), indicating varied initial muscle responses. Positive stimuli show increased variability at the beginning and end, possibly indicating increased muscle activity during these times. In contrast, a stable EMG response is seen with the negative stimulus, which maintains a consistent amplitude across segments. These variations provide insight into the different muscle responses and time dynamics for the different interventions.

The analysis of EMG signal amplitude across stimuli for the right Sternocleidomastoid muscle indicates clear differences. The silence stimulus elicits a high mean amplitude and a wide dynamic range, indicating increased muscle activity. In contrast, the music stimulus shows a significantly lower mean amplitude and a narrower range, suggesting reduced muscle activation compared to the others. The positive and negative interventions fall in between, with moderate mean amplitudes and intermediate ranges. A segment-wise exploration provides additional details: Segment 1 of the silence intervention shows a high amplitude and high variability, while Segment 3 suggests increased muscle activation. In the music intervention, segment 5 highlights variability, while segment 6 of the positive stimulus indicates significant activity. For negative stimuli, segment 1 shows pronounced variation and substantial range, and segment 2 shows higher variability. These different segment characteristics across the interventions likely reflect different muscular engagements and responses.

Both muscles show fluctuations in activity across segments during the different interventions, indicating temporal variations in muscular responses. However, there are clear differences in their mean amplitudes and dynamic ranges across stimuli. For example, during the silence stimulus, the left muscle tends to have lower mean amplitudes compared to the right, suggesting potentially increased activity in the latter. In addition, certain segments show different levels of variability or mean amplitude between the left and right muscles within the same intervention, indicating nuanced responses to stimuli. Despite these differences, both muscles show consistent patterns within themselves, suggesting underlying physiological factors that influence their responses to different stimuli.

### 3.2. Post Hoc Analysis of Extracted Features across Four Muscle Sites

#### 3.2.1. Analysis of Sternocleidomastoid Muscle (SCM)

The post hoc *t*-tests conducted on the time point pairs derived from EMG signals of the SCM yielded distinct outcomes for both the left and right sides and are shown in Table 5. In the left SCM, feature SF distinguished T1 and T6 (*p* = 0.02) and T2 and T6 (*p* = 0.025), suggesting its potential as a temporal discriminator. Conversely, the right SCM showed a more intricate pattern of feature variations. Features MO, CO, CH, and HZ exhibited significant differentiation (*p* < 0.05) across most time points but not for the T3–T4, T4–T5, and T5–T6 intervals. This pattern suggests potential functional similarities or subtle fluctuations during these specific periods in the right SCM. Notably, intervention pairs did not elicit significant differences in either muscle side. These results highlight unique patterns in the right SCM. These insights are valuable for further elucidating SCM function and its relationship to various interventions.

**Figure 5:**
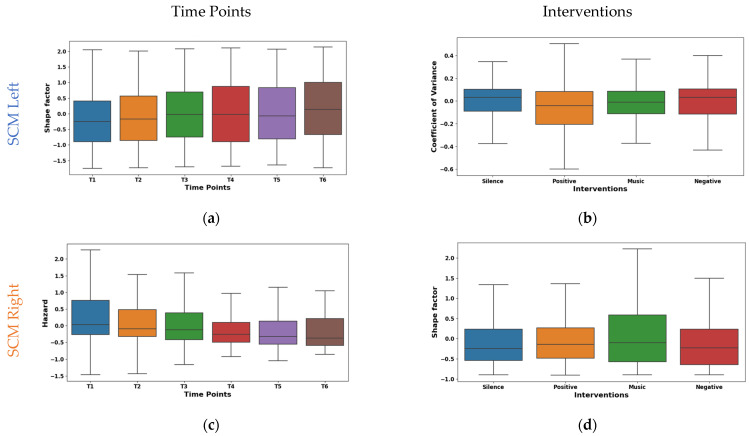
Boxplot illustrating key features for distinguishing time points (**a**,**c**) and interventions (**b**,**d**) in the SCM.

The shape factor in Figure 5a exhibited distinct temporal dynamics in EMG signals from the left SCM across time points T1–T6. It was observed that the central tendency shifted significantly across time points. The mean values shifted from negative (~−0.149 at T1) to positive (~0.167 at T6), with a prominent increase at T6. The median followed a similar trend, reaching 0.242 at T6. The interquartile range (IQR) remained consistent except for T4, suggesting varying data dispersion. These shifts indicate potential muscle activity pattern variations. The right SCM hazard feature shown in Figure 5c exhibited a marked temporal decline across time points. The mean values significantly decreased from 0.308 at T1 to −0.173 at T6, with the median mirroring this trend (−0.002 to −0.391). While IQR varied across time points, T5–T6 showed reduced dispersion, suggesting a more consistent muscle activity pattern.

The left SCM coefficient of variance varied across interventions, as shown in Figure 5b. During silence, minimal rapid activation changes (mean −0.015, median 0.032) were observed, suggesting relaxation. The negative intervention showed potentially quicker and synchronized recruitment (mean 0.052, median 0.032). This may be due to heightened stress induced by auditory stimulation. From the right SCM shape factor boxplot illustrated in Figure 5d, it was found that the silence intervention and negative reinforcement stimulation showed a low amplitude and irregular recruitment (means −0.009, −0.169). Whereas, with music stimulation, a higher amplitude and phasic patterns (mean 0.173) were observed, which may be due to the increased alertness. However, the positive reinforcement (mean ~−0.03, median ~−0.04) showed an intermediate pattern in both the left and right SCM, suggesting context-dependent adjustments.

#### 3.2.2. Analysis of Cervical Erector Muscle (CEM)

The post hoc *t*-tests in Table 6 reveal significant variations (*p* < 0.05) within the left SCM for features DR, MA, and MO. All three features differed significantly between T3 and T4 (*p* = 0.04 for DR and MA, *p* = 0.01 for CO), and DR and MA also differed between T3 and T6 (*p* = 0.04). These findings suggest specific time intervals, particularly around T3, exhibiting distinct patterns or changes in left SCM activity. Notably, no such significant differences were observed in the right SCM, indicating potential functional asymmetry between the muscles. The right CEM can differentiate M&P, N&S, and P&S interventions with *p* < 0.05.

**Figure 6:**
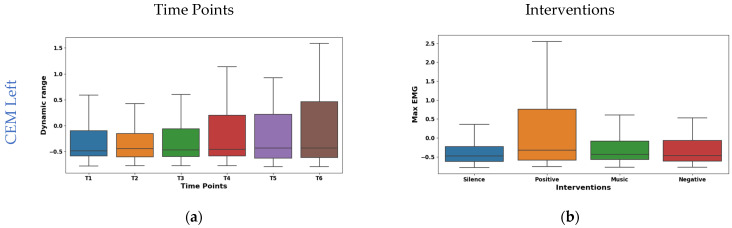

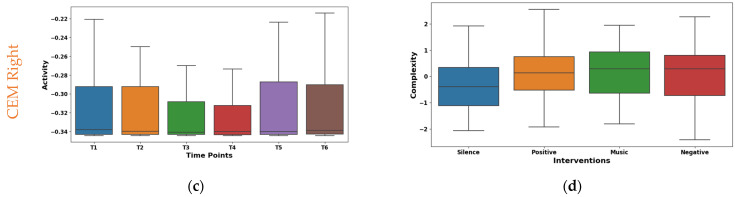
Boxplot illustrating key features for distinguishing time points (**a**,**c**) and interventions (**b**,**d**) in the CEM.

The boxplot in Figure 6a shows the temporal analysis of the dynamic range feature extracted from EMG signals of the left CEM. Notably, the mean values shifted from negative at the initial time points (−0.049 at T1, −0.059 at T2) to positive at later points (0.070 at T4, 0.056 at T5), slightly decreasing to 0.027 at T6. However, the median is consistent around −0.15 for all time points. This suggests potential asymmetry in the distribution of dynamic range values. Interestingly, IQR exhibited a consistent increase from 1.408 at T1 to 2.037 at T6, reflecting a wider range of muscle activity patterns. As shown in Figure 6c, the right CEM Hjorth activity exhibited dynamic temporal fluctuations: the mean oscillated (−0.075 to 0.061), but the median remained stable (−0.34). The IQR values initially ranged from 0.050 to 0.030 and then fluctuated across the time points. This implies dynamic changes in data spread across the time points. These findings highlight Hjorth Activity’s temporal sensitivity and potential for capturing dynamic muscle activity patterns.

The interventions elicited varied maximum amplitude responses in the left CEM (shown in Figure 6b). The highest mean (0.266) was found during positive reinforcement stimulation. This suggests increased left CEM activation for a positive effect. Whereas, silence had the lowest mean (−0.206), possibly indicating reduced activation due to relaxation. From the box plot of right CEM Hjorth complexity shown in Figure 6d, it was found that silence (mean −0.369, median −0.395) showed the lowest complexity, possibly reflecting relaxed control. Whereas the positive intervention (mean 0.163, median 0.136) showed the highest complexity, potentially indicating increased motor unit coordination or nuanced control. Further, the music and negative interventions showed an intermediate effect in both the left and right CEM.

#### 3.2.3. Analysis of Quad Muscle (QM)

Table 7 shows the post hoc analysis of QM features, revealing significant differences among various time point pairs. It was observed that the left QM exhibited considerable temporal variations. Feature CO exhibited numerous significant pairwise differences with time points T2 (*p* = 0.016), T3 (*p* = 0.002), T4 (*p* = 0.008), T5 (*p* = 0.01), and T6 (*p* = 0.04), suggesting a pronounced shift in neuromuscular activity post T1. Also, a significant difference was found between time point pair T2 & T3 (*p* = 0.03). Additionally, feature CH showed a significant distinction between T1 & T3 (*p* = 0.001), T1 & T4 (*p* = 0.03), and T2 & T3 (*p* = 0.008). This pattern suggests a potential step change in left QM activation following the initial stimulus represented at T1. These differences are crucial for understanding psychological responses and developing targeted interventions. CO exhibits greater temporal variability compared to CH, suggesting a more dynamic pattern of change across time points. Similar to the CEM, the right QM also did not exhibit any statistically significant differences. Post hoc analyses of right and left QM activity revealed feature-specific differences in intervention effects. The left QM’s MF distinguished the M & S (*p* = 0.03), while the right QM’s CO differentiated the P & S interventions (*p* = 0.03). These findings suggest a nuanced interplay between intervention type, laterality, and specific neural markers within the TM.

**Figure 7:**
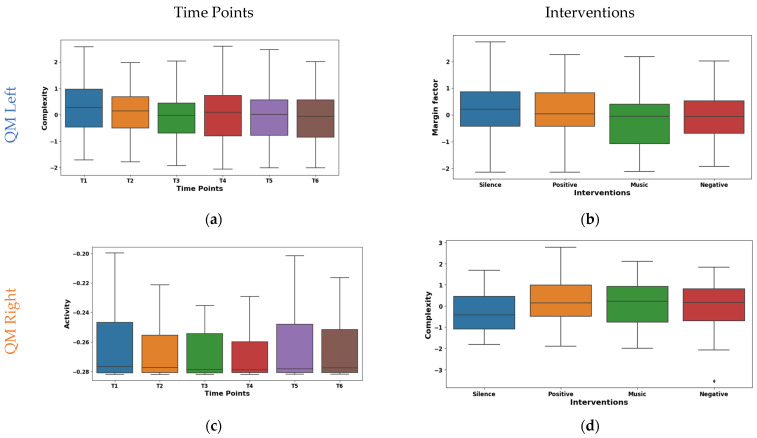
Boxplot illustrating key features for distinguishing time points (**a**,**c**) and interventions (**b**,**d**) in the QMs.

The temporal analysis of Hjorth complexity in EMG signals from the left QM shown in Figure 7a demonstrated a notable downward trend across time points. The mean values declined from 0.278 at T1 to −0.106 at T5, with a slight rebound to −0.086 at T6. The potential shift in the mean values from high/complex activity (T1–T3) to lower/focused activity (T4–T5) may be due to fatigue. It was observed that the median also exhibited a similar pattern of shifting from 0.274 at T1 to −0.059 at T6. However, IQR fluctuated across time points, with an initial decrease from 1.435 at T1 to 1.181 at T2, followed by a gradual increase to 1.520 at T4 and a subsequent decline to 1.415 at T6. Right QM Hjorth activity exhibited fluctuating mean values across T1–T6, as visualized in the box plot of Figure 7c. The mean values oscillated between 0.086 at T1 and −0.075 at T3, with a mean of 0.0004 at T4 and −0.048 at T5. The median values emerged at T5 (0.007) and increased to 0.189 at T6. This suggests a potential shift from high-complexity, diverse muscle activation to lower-complexity, potentially fatigued patterns.

The margin factor box plot in Figure 7b reveals diverse responses in left QM activity across the different interventions. It was found that silence and positive reinforcement (means 0.121, 0.134) showed higher amplitude and potentially more phasic motor unit activation patterns. Whereas, during the music intervention (mean −0.216), a lower-amplitude and possibly tonic patterns were observed. Further, the negative reinforcement demonstrated an intermediate mean (−0.039) and median (−0.060). The box plot of right QM Hjorth complexity shown in Figure 7d varied across interventions. The highest complexity was observed during the silence intervention (mean 0.199, median 0.290). This may be due to the enhanced motor unit coordination in the silence intervention. Whereas, the positive reinforcement and music interventions were intermediate. These findings highlight the differential neuromodulatory influences of the interventions on QM activity.

#### 3.2.4. Analysis of Tibialis Muscle (TM)

The post hoc *t*-tests for the left TM features illustrated in Table 8 revealed significant temporal variations in features CV, MO, CO, CH, and HZ. CV exhibited significant pairwise differences in the early time points (T1–T5), with *p* < 0.01. Additionally, feature MO showed a biphasic pattern, showcasing significant variance across both early (T1–T5) and late (T2–T6, T4–T6) time points, with *p*-values satisfying both stringent and moderately stringent criteria (*p* < 0.01 and *p* < 0.05). Features CO and CH primarily differed from T1, with significant contrasts observed at later stages (T5 and T6 for CO; T1–T6 for CH), all *p* < 0.01. Conversely, HZ exhibited significant differences at later time points (T1–T6, T2–T5), with *p* < 0.01. These findings suggest diverse temporal profiles for each feature, underscoring the intricate interplay between time and their respective trajectories. Our analysis revealed substantial temporal dynamics within right TM activity, characterized by significant modulations in multiple features (*p* < 0.05). Notably, Features MO, CO, CH, and HZ exhibited extensive differentiation across all pairwise time point comparisons (*p* < 0.05) except for T5 & T6. This suggests highly intricate temporal variations among the features. CV demonstrated distinct patterns at earlier time points, differentiating T1 from T3, T5, and T6 and mirroring these effects for T2 & T3, T5, and T6 (*p* < 0.01 for T1–T3; *p* < 0.006 for T2–T3). SF showed distinguishing ability at later time points, distinguishing T1 from T5 and T6 and replicating this at T2 vs. T5 and T6 (*p* < 0.03). Interestingly, MF remained invariant across time points, suggesting a stable underlying process. These findings highlight the complex interplay between time and specific neural signatures within right TM activity.

**Figure 8:**
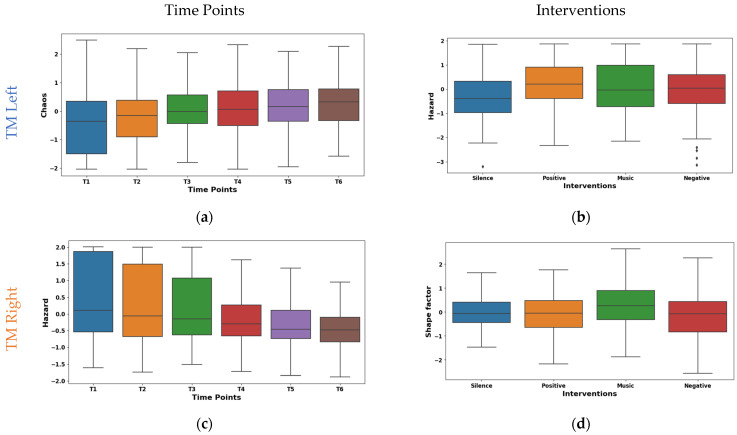
Boxplot illustrating key features for the distinguishing time points (**a**,**c**) and interventions (**b**,**d**) in the TM.

The box plot of left TM Hjorth chaos shown in Figure 8a exhibits a substantial upward trend across time points. The mean values ascended from −0.403 at T1 to 0.290 at T6. This suggests a progressive shift towards more chaotic and unpredictable muscle activity patterns. Whereas the median values mirrored this trend by rising from −0.353 at T1 to 0.326 at T6. Notably, the IQR values ranged from 1.104 to 1.839, suggesting a moderate spread of Hjorth chaos values within the muscle. The box plot shown in Figure 8c represents our temporal analysis of Hjorth hazard in the EMG signals from the right TM; a notable upward trajectory across time points is demonstrated. This indicates a progressive shift towards more rapid and abrupt signal changes. The mean values ascended from −0.471 at T1 to 0.383 at T6. A similar pattern is shown by the median values rising from −0.193 to 0.401. Concurrently, IQR exhibited a consistent decline from 2.168 at T1 to 0.810 at T6. These potential transitions in muscle activation patterns may be indicative of enhanced muscle responsiveness or fatigue-induced adaptations.

The box plot of interventions illustrated in Figure 8b differentially modulated Hjorth hazard in the left TM. The lowest mean (−0.143) was observed during the silence intervention, which reflects a more relaxed or sustained muscle activation pattern. Conversely, the music, positive, and negative interventions demonstrated positive means (0.021, 0.072, and 0.051). This may be due to the increased rapid signal shifts that reflect more dynamic or phasic muscle activation strategies. Similar to the left TM, as shown in Figure 8d, the interventions had different effects on shape factor in the right TM. The highest mean (0.245) and median (0.254) was observed during the music intervention. This may be due to the shift towards a higher amplitude. Conversely, negative reinforcement exhibited the lowest mean (−0.192), and positive reinforcement showed the lowest median (−0.052). This indicates the lower amplitudes that are reflected by the tonic muscle activation strategies. Furthermore, an intermediate mean (0.011) and median (−0.069) values were found during the silence intervention. These findings highlight the diverse neuromodulatory influences of the interventions on TM activity patterns.

### 3.3. Classification of Psychological Interventions

The classification performance of the RF machine learning algorithm when analyzing EMG signals for classifying psychological interventions is shown in Table 9. It was observed that the TM consistently yielded the highest performance in all metrics. Notably, the left TM yielded the highest performance (Acc = 63.20%, Rec = 66.36%, F1 = 63.34%, Spe = 75%, AUC = 84.61%). The SCM exhibited moderate performance in classifying psychological interventions, with an F1 of 40% to 42% for both the left and right sides. Particularly, the CEM exhibited a substantial difference in performance between the left (higher Acc, Pre, Rec, and F1 = ~57–58%) and right sides (lower Acc, Pre, Rec, and F1 = ~37–40%). Similar to the CEM, the QM demonstrates higher performance on the left side (F1 = 46.77%) compared to the right (F1 = 32%).

## 4. Discussion

To the authors’ knowledge, this is the first study aimed at understanding how various auditory stimuli may influence motor activation patterns during overground walking. Although significant evidence exists suggesting inter-individual motor activation patterns during overground walking, there is also evidence suggesting that there is no intra-individual variability in walking gait [6,7,8]. Our findings indicate that auditory stimuli may illicit unique muscle activation responses in the Sternocleidomastoid (SCM), Cervical Erector (CEM), Quadriceps (QM), and Tibialis (TM). These findings add significantly to the literature as these results suggest that auditory psychological interventions may indeed modify human movement through changes in muscle activation patterns, which may have significant implications in rehabilitation, sports science, ergonomics, and healthcare. Taken together, our findings provide evidence that auditory cues may cause changes in motor control and potentially impact performance outcomes.

In our study, the participants walked in the same direction and made left turns only suggesting that similar motor activation patterns would be noted between conditions. However, the complexity of the sEMG signals of the right CEM were significantly different between the silence intervention and the other three interventions. These findings suggest that during the silence intervention, the muscle activity intensity, activation changes, and movement of the CEM were different from how they were in the other three interventions. When the CEM unilaterally contracts, it causes ipsilateral flexion of the neck, and our findings suggest that silence may cause the right CEM increase motor unit coordination or nuanced control during the silence condition. Interestingly, these findings were also noted in the QM, as the right and left QM were found to have distinct responses to silence compared to music (left QM) and positive cues (right QM). These findings support the work of previous scholars who have sought to disrupt the neurobiomechanical control of the trunk and limbs during locomotion [13,14,15]. They may also further the work of Rumsey and colleagues [26], who reported that there was greater muscle activation in their anxiety group. Although we did not analyze mood states in this study, our findings suggest that silence may disrupt muscle activity patterns; however, the unilaterality of our results for both CEM and QM are quizzical and must be explored further.

The other interesting findings of this study were the asymmetrical changes in muscle activation patterns over time for all the different muscle groups. While differences in motor activation patterns between each side were expected as the participants were instructed to turn left only while walking, the asymmetrical changes over time are quizzical. For example, our findings suggest that there is a significantly higher complexity in left QM activation during the first 5 min compared to all other time points, while T2 also had significantly greater complexity than T3. These changes are in line with those noted in the left TM, as the left TM has higher complexity over time. These changes suggest that participants may have been using compensatory strategies during the first 10 min of walking by quickly adjusting their gait, and over time, these strategies were reduced [40]. However, the unilaterality of these changes in the QM but not in TM are quizzical. One potential explanation might be the fact that the participants turned to their left, and the changes in left QM complexity may be attributed to changes in turning strategies over time, while the bilateral changes in TM complexity may be attributed to shifts in walking strategy over time [41]. The same explanation may also support the findings of the data from the SCM, as the SCM is responsible for turning the head in the opposite direction, and the fluctuations in the right SCM may be explained by participants seeking to optimize turning strategies over the course of the 30 min walk.

The findings of this study have significant implications for rehabilitation professionals, coaches, strength and conditioning coaches, and other individuals who seek to optimize movement performance. We find that these practitioners must be aware that word choices (e.g., negative feedback vs. positive feedback) or whether the patient or athlete chooses to exercise in silence or while listening to music may have implications on muscle activation patterns. Further, these findings support the fact that participants are constantly adjusting walking strategies and that it may be challenging to obtain true biometric data from muscle activation patterns in gait as humans are constantly adjusting these strategies to smoothen their walking gait.

Employing a multi-feature approach with sEMG revealed a heterogeneous landscape of the best separating features for the time point pairs and interventions. Both the statistical and Hjorth features emerged as potent discriminators, with a distribution suggesting diverse adaptations in muscle recruitment (shape factor, hazard), activation intensity (dynamic range, activity), neuromuscular control (complexity, margin factor), and signal dynamics (chaos). The identified features hold promises as potential markers for monitoring muscle function across various clinical and research applications. Further RF classification of muscle-specific interventions revealed heterogeneous performance across muscle sites, with the TM exhibiting the highest accuracy and AUC.

This study is not without limitations. The primary limitation of this study is the fact that there is significant inter-individual variability in walking gait [13,14,15], and significant variability was observed in our subjects. However, by adopting a within-person design, we aimed to mitigate the influence of this variability in our analyses. Another potential limitation of this study is that the study was originally powered for measuring mood effects and may have not been correctly powered for this analysis. Finally, while this study provides significant evidence of changes in muscle activation patterns over time and between interventions, the use of a less conservative correction technique (i.e., Benjamini–Hochberg False Detection Rate), we may have reported more significant findings.

## 5. Conclusions

The present study provides compelling evidence for the modulation of muscle activation patterns by auditory stimuli, showcasing the potential of employing wearable EMG sensors and feature-rich analysis for differentiating interventions and identifying muscle-specific biomarkers. The dynamic and nuanced responses observed in muscles such as the SCM, CEM, and TM, particularly within specific time periods and intervention pairs, highlight the complex interplay between auditory input, cognitive processing, and motor control. Moreover, the robust performance of the feature-based RF classification carried out in this study, especially for the TM, paves the way for personalized neuroadaptive interventions tailored to individual responses and muscle recruitment patterns. Future research should focus on investigating the underlying neural mechanisms of these modulations and exploring the clinical applications of this approach in enhancing rehabilitation, optimizing athletic performance, and promoting well-being across diverse patient populations and contexts.

## Figures and Tables

**Table 1:** Demographic characteristics of the participants.

Characteristic	N
Total subjects	36
Excluded subjects	10
Gender	16 females
Height	167.32 ± 20.20 cm
Weight	69.93 ± 9.76 kg
Age	23.04 ± 5.64 years

**Table 2:** List of positive and negative interventions used in the study.

Positive Reinforcement	Negative Reinforcement
Good job, you’re doing awesome!	You’ve got to walk faster than that.
Keep up the good work!	You’re so slow!
You’ve got this!	Why do you walk like that?
You’re almost done, just a few more minutes!	Did you learn how to walk yesterday?
That’s a great pace!	You’re doing terrible.
You’re going strong! Keep it up!	Who walks like that?
Nice work.	You have potential but you don’t use it.
Great job!	You’ll never amount to anything.
Good stuff. Keep it up.	You’re not putting very much effort into this.
You’re doing an amazing job.	This is the worst pace you’ve had yet.

**Table 3:** List of statistical and Hjorth features extracted from the EMG signals.

Feature	Expression	Description
Coefficient of variance (CV) [31]	$\frac{\frac{1}{N}\sum_{n=1}^{N}x_{i}\left[n\right]}{\sqrt{\frac{1}{N}{\sum_{n=1}^{N}\left[x_{i}\left[n\right]-f_{1}\right]}^{2}}}$	Measures relative variability, indicating how spread out values are compared to the mean.
Shape factor (SF) [31]	$\frac{\sqrt{\frac{1}{N}{\sum_{n=1}^{N}\left[x_{i}\left[n\right]\right]}^{2}}}{\frac{1}{N}\sum_{n=1}^{N}\left|x_{i}\left[n\right]\right|}$	Captures signal shape characteristics, often reflecting muscle contraction patterns.
Margin factor (MF) [31]	$\frac{\max\left|x_{i}\right|}{{\left(\frac{1}{N}\sum_{n=1}^{N}\left|\left[x_{i}\left[n\right]\right]\right|\right)}^{2}}$	Quantifies signal margin, representing the proportion of time a signal is active.
Dynamic range (DR) [32]	$\max(x_{i}\left[n\right])-\max(x_{i}\left[n\right])$	Reflects the signal’s amplitude range, capturing its highest and lowest values.
Max amplitude (MA) [32]	$\max(x_{1},x_{2},x_{3}\ldots x_{N})$	Represents the signal’s maximum amplitude, indicating its peak intensity.
Activity (AC) [33]	$\frac{1}{N}\sum_{i=1}^{N}{\left(X_{i}-mean(X_{i})\right)}^{2}$	Quantifies muscle activation intensity.
Mobility (MO) [33]	$\frac{AC\left(x^{\prime }\left(n\right)\right)}{AC\left(x\left(n\right)\right)}$	Indicates muscle activation changes and movement.
Complexity (CO) [33]	$\frac{MO\left(x^{\prime }\left(n\right)\right)}{MO\left(x\left(n\right)\right)}$	Combines activity and mobility indicators.
Chaos (CH) [33]	$\frac{CO\left(x^{\prime }\left(n\right)\right)}{CO\left(x\left(n\right)\right)}$	Assesses irregular muscle activation patterns.
Hazard (HZ) [33]	$\frac{CH\left(x^{\prime }\left(n\right)\right)}{CH\left(x\left(n\right)\right)}$	Measures sudden muscle activation shifts.

**Table 4:** Description of the performance metrics of the classifier used in the study.

Metric	Measure
Acc	Overall performance of the model in correctly classifying both positive and negative cases.
Pre	The model’s ability to avoid false positives, ensuring that positive predictions are highly likely to be correct.
Rec	The model’s ability to detect true positives, ensuring that most actual positive cases are captured.
F1	A balanced assessment of both precision and recall, capturing both the model’s ability to avoid false positives and its ability to detect true positives.
Spe	The model’s ability to avoid false negatives, ensuring that negative predictions are highly likely to be correct.
AUC	The model’s ability to distinguish between classes across all possible thresholds, representing its overall discriminative power.

**Table 5:** Statistical significance of the time points and intervention pairs analyzed via a post hoc *t*-test in the SCM.

		Timepoint Pairs															Intervention Pairs					
	Pairs	T1 & T2	T1 & T3	T1 & T4	T1 & T5	T1 & T6	T2 & T3	T2 & T4	T2 & T5	T2 & T6	T3 & T4	T3 & T5	T3 & T6	T4 & T5	T4 & T6	T5 & T6	M & N	M & P	M & S	N & P	N & S	P & S
Features																						
CV																						
SF						**L**				**L**												
MF																						
DR																						
MA																						
AC																						
MO		**R**	**R**	**R**	**R**	**R**	**R**	**R**	**R**	**R**			**R**		**R**							
CO		**R**	**R**	**R**	**R**	**R**	**R**	**R**	**R**	**R**			**R**		**R**							
CH		**R**	**R**	**R**	**R**	**R**	**R**	**R**	**R**	**R**		**R**	**R**									
HZ		**R**	**R**	**R**	**R**	**R**		**R**	**R**	**R**		**R**										

**L** and **R** represents statistical significance (*p* < 0.05). **L** = Significant for Left muscle, **R** = Significant for Right muscle.

**Table 6:** Statistical significance of the time points and intervention pairs analyzed via a post hoc *t*-test in the CEM.

		Timepoint Pairs															Intervention Pairs					
	Pairs	T1 & T2	T1 & T3	T1 & T4	T1 & T5	T1 & T6	T2 & T3	T2 & T4	T2 & T5	T2 & T6	T3 & T4	T3 & T5	T3 & T6	T4 & T5	T4 & T6	T5 & T6	M & N	M & P	M & S	N & P	N & S	P & S
Features																						
CV																						
SF																						
MF																						
DR											**L**		**L**									
MA											**L**		**L**									
AC																						
MO											**L**											
CO																			**R**		**R**	**R**
CH																						
HZ																						

**L** and **R** represents statistical significance (*p* < 0.05). **L** = Significant for Left muscle, **R** = Significant for Right muscle.

**Table 7:** Statistical significance of the time points and intervention pairs analyzed via a post hoc *t*-test in the QMs.

		Timepoint Pairs															Intervention Pairs					
	Pairs	T1 & T2	T1 & T3	T1 & T4	T1 & T5	T1 & T6	T2 & T3	T2 & T4	T2 & T5	T2 & T6	T3 & T4	T3 & T5	T3 & T6	T4 & T5	T4 & T6	T5 & T6	M & N	M & P	M & S	N & P	N & S	P & S
Features																						
CV																						
SF																						
MF																			**L**			
DR																						
MA																						
AC																						
MO																						
CO		**L**	**L**	**L**	**L**	**L**	**L**															**R**
CH			**L**	**L**			**L**															
HZ																						

**L** and **R** represents statistical significance (*p* < 0.05). **L** = Significant for Left muscle, **R** = Significant for Right muscle.

**Table 8:** Statistical significance of the time point and intervention pairs analyzed via a post hoc *t*-test in the TM.

		Timepoint Pairs															Intervention Pairs					
	Pairs	T1 & T2	T1 & T3	T1 & T4	T1 & T5	T1 & T6	T2 & T3	T2 & T4	T2 & T5	T2 & T6	T3 & T4	T3 & T5	T3 & T6	T4 & T5	T4 & T6	T5 & T6	M & N	M & P	M & S	N & P	N & S	P & S
Features																						
CV		**L**	**L** **R**	**L**	**L** **R**	**R**	**L** **R**	**L**	**L** **R**	**R**												
SF					**R**	**R**			**R**	**R**		**R**	**R**									
MF																						
DR		**R**			**R**	**R**				**R**												
MA					**R**	**R**																
AC		**R**		**R**	**R**	**R**			**R**													
MO		**L** **R**	**L** **R**	**L** **R**	**L** **R**	**L** **R**	**R**	**R**	**L** **R**	**L** **R**	**R**	**R**	**R**	**R**	**L** **R**							
CO		**L** **R**	**L** **R**	**L** **R**	**L** **R**	**L** **R**	**R**	**R**	**L** **R**	**L** **R**	**R**	**R**	**R**	**R**	**R**							
CH		**L** **R**	**L** **R**	**L** **R**	**L** **R**	**L** **R**	**L** **R**	**L** **R**	**L** **R**	**L** **R**	**R**	**L** **R**	**L** **R**	**R**	**L** **R**							
HZ		**L** **R**	**L** **R**	**L** **R**	**L** **R**	**L** **R**	**R**	**R**	**L** **R**	**L** **R**	**R**	**R**	**R**	**R**	**R**							

****L**** and ****R**** represents statistical significance (*p* < 0.05). ****L**** = Significant for Left muscle, ****R**** = Significant for Right muscle.

**Table 9:** Performance assessment of Random Forest in classifying muscle-specific interventions.

		Accuracy	Precision	Recall	F1-Score	Specificity	AUC
SCM	Left	39.20	44.09	39.20	40.09	53.33	65.66
	Right	41.60	46.67	41.60	42.38	57.14	65.23
CEM	Left	56.80	59.55	56.80	57.26	82.35	77.40
	Right	37.60	39.41	37.60	37.70	75.00	63.10
QM	Left	46.40	47.82	46.40	46.77	70.59	74.98
	Right	32.00	35.04	32.00	32.55	69.23	64.66
TM	Left	63.20	64.42	63.20	63.34	75.00	84.61
	Right	60.80	66.36	60.80	61.30	73.68	84.56

## Data Availability

Due to IRB restrictions, data can be obtained from author A.B.
